# Microbial Allies or Adversaries? The Genotype-Dependent Impact of Inoculation on Silver Birch

**DOI:** 10.3390/plants14040545

**Published:** 2025-02-10

**Authors:** Greta Striganavičiūtė, Dorotėja Vaitiekūnaitė, Milana Šilanskienė, Vaida Sirgedaitė-Šėžienė

**Affiliations:** Laboratory of Forest Plant Biotechnology, Institute of Forestry, Lithuanian Research Centre for Agriculture and Forestry, LT-53101 Kaunas, Lithuania; doroteja.vaitiekunaite@lammc.lt (D.V.); milana.silanskiene@lammc.lt (M.Š.); vaida.seziene@lammc.lt (V.S.-Š.)

**Keywords:** plant–microbe interactions, biochemical markers, environmental contaminants, Betula pendula

## Abstract

Microbial inoculation plays a crucial role in shaping plant physiological and biochemical responses, influencing growth, secondary metabolism, and stress-related markers. This study investigates the effects of PAH-degrading microorganisms (*Pseudomonas putida*, *Sphingobium yanoikuyae*, and *Rhodotorula sphaerocarpa*) on the growth, secondary metabolism, photosynthetic pigment, and stress-related biochemical markers of silver birch (*Betula pendula* Roth) seedlings from two half-sib families grown hydroponically. Results demonstrate family-dependent variations in the response to microbial treatments. In family 73, the growth of both shoots and roots was inhibited by certain microbial treatments, along with a decrease in key biochemical markers such as phenolic content and carotenoids. Conversely, family 86 showed no growth inhibition and exhibited improvements in some biochemical markers, including flavonoids and chlorophyll. Stress indicators, such as malondialdehyde (MDA) and soluble sugars, displayed contrasting patterns between families, with increased MDA observed in family 73 under certain microbial treatments. In contrast, family 86 did not exhibit an increase in MDA, suggesting differences in stress mitigation. Soluble sugars were generally reduced in family 73. Antioxidant enzyme activity further highlighted these family-specific responses, with variations in enzymes like ascorbate peroxidase (APX) and guaiacol peroxidase (POX) across treatments. Notably, significant interactions between family and microbial treatments were observed for several oxidative stress enzymes, underscoring the role of genotype in shaping the response to microbial stress. These findings highlight the genotype-dependent interactions between microbial inoculation and plant secondary metabolism, providing insights into the role of specifically selected microbial inoculation in stress mitigation and growth regulation.

## 1. Introduction

Microbial inoculation has emerged as a promising strategy to enhance plant growth, improve stress resilience, and support sustainable ecosystem management. Beneficial microbes, including bacteria and fungi, can establish symbiotic or associative interactions with plants, conferring numerous advantages such as improved nutrient acquisition, modulation of plant hormone levels, and protection against abiotic stressors. These interactions play a crucial role in plant health by enhancing root development, increasing water and nutrient uptake, and inducing systemic resistance mechanisms that bolster plant defense responses [1,2,3,4,5].

Abiotic stress factors, including heavy metals, drought, salinity, and organic pollutants, pose significant challenges to plant growth and survival. Among these, polycyclic aromatic hydrocarbons (PAHs) are particularly concerning due to their persistence, toxicity, and hydrophobic nature, which hinder their natural degradation in the environment [6,7,8,9]. Traditional remediation methods are often costly and environmentally invasive, making biological approaches such as phytoremediation more attractive. Phytoremediation, a plant-based remediation strategy, capitalizes on the natural ability of plants to tolerate, accumulate, and transform organic pollutants while fostering beneficial microbial activity in the rhizosphere [9,10,11,12,13,14,15].

Microbial inoculants have demonstrated potential in mitigating pollutant-induced stress by modulating plant physiological and biochemical responses [5,16,17,18,19]. Certain rhizobacteria and endophytic fungi can enhance growth, enhance antioxidant enzyme activities, reduce oxidative damage, promote secondary metabolite production, and influence the synthesis of phytohormones, which regulate plant growth and stress adaptation, contributing to improved stress tolerance. For example, endophytic fungi *Penicillium roqueforti* Thom. was shown to significantly enhance the resistance of wheat plants to heavy metal stress by limiting metal uptake from the soil. The fungus achieved this by secreting indole-3-acetic acid (IAA), which contributed to improved root development and enhanced stress tolerance. Furthermore, wheat seedlings inoculated with *P. roqueforti* and irrigated with wastewater exhibited superior growth, increased nutrient uptake, and reduced heavy metal accumulation in both roots and shoots. In contrast, non-inoculated wheat plants exposed to heavy metals exhibited stunted growth and symptoms of chlorosis [5]. Similarly, willow inoculation with *Sphingobium yanoikuyae* strain promoted Cd accumulation in the roots and mitigated Cd toxicity by regulating root growth, activating the antioxidant enzyme system, and remodeling cell wall polysaccharides. Under Cd stress, this bacterium significantly increased root length and biomass, elevated root IAA levels, and enhanced Cd retention in the cell walls. This localized sequestration of Cd, combined with enhanced catalase activity, resulted in reduced hydrogen peroxide (H_2_O_2_) and malondialdehyde (MDA) levels, thereby improving Cd tolerance in *Salix matsudana* roots. Importantly, the inoculation did not alter root cell wall structure or induce oxidative stress, indicating a stable and beneficial symbiotic relationship between the bacterium and *S. matsudana* [20].

These beneficial microbes also play a crucial role in direct PAH degradation by breaking down complex hydrocarbons into less harmful compounds, thus reducing toxicity in the substrate [21,22].

The interactions between trees and microbial inoculants remain an area of ongoing research, particularly in species with high ecological adaptability. Silver birch (*Betula pendula* Roth.), a pioneer tree species known for its resilience to diverse environmental conditions, serves as an excellent model for studying plant–microbe interactions under stressful conditions [23,24]. Previous studies have identified microbial species, including *Pseudomonas* spp., *Sphingobium* spp., and *Rhodotorula* spp., as potentially effective degraders of PAHs [25,26,27,28]. However, the specific mechanisms by which these microbes influence tree physiology and biochemical pathways require further investigation. Understanding these mechanisms is essential for developing targeted microbial applications to improve tree establishment and growth in contaminated landscapes.

This study examines the effects of PAH-degrading microbial inoculation with *Pseudomonas putida* Trevisan, *Sphingobium yanoikuaye* Yabuuchi et al., and *Rhodotorula sphaerocarpa* (S.Y. Newell and Fell) Q.M. Wang, F.Y. Bai, M. Groenewald and Boekhout on the growth and biochemical responses of silver birch seedlings under controlled hydroponic conditions. By evaluating key physiological and enzymatic indicators, such as antioxidant enzyme activities and secondary metabolite production, this research aims to elucidate the role of beneficial microbes in promoting plant resilience. The findings contribute to a broader understanding of plant–microbe interactions and offer insights into the potential applications of microbial inoculants in phytoremediation and sustainable forestry. Additionally, this study provides a foundation for integrating microbial technologies into afforestation and reforestation efforts, ensuring the establishment of resilient tree populations in contaminated and degraded environments.

## 2. Results

### 2.1. Seedling Growth and Development

**Figure 1:**
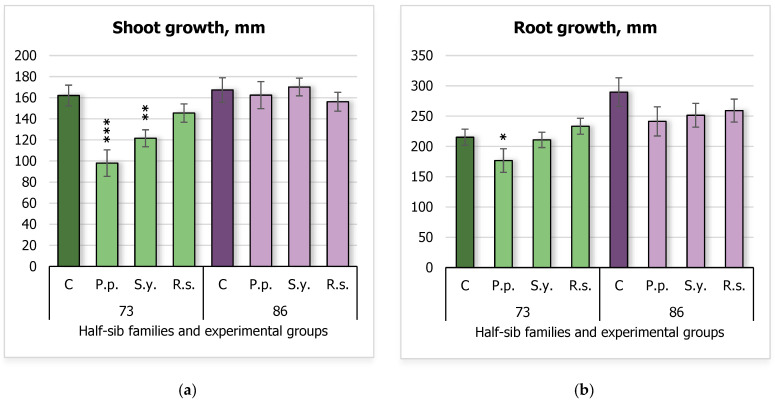
Comparison of shoot growth (mm) (**a**) and the longest root length (**b**) in silver birch half-sib families (73 and 86) under different bacterial treatments: control (C), *Pseudomonas putida* (*P.p.*), *Sphingobium yanoikuyae* (*S.y.*), and *Rhodotorula sphaerocarpa* (*R.s.*). Error bars represent the standard error (SE). Statistically significant differences compared to the control group were assessed using the Kruskal–Wallis H test: * *p* < 0.05; ** *p* < 0.01; *** *p* < 0.001.

During this study, the impact of PAH-degrading bacteria on silver birch seedling growth was assessed, focusing on shoot length (Figure 1a) and the longest root length (Figure 1b). Statistically significant effects were observed in only one of the two tested half-sib families, family 73. Shoot growth (Figure 1a) was reduced in seedlings inoculated with *Pseudomonas putida* (*P.p.*) and *Sphingobium yanoikuyae* (*S.y.*), with the control group (C) showing a mean shoot length of 162.1 ± 9.84 mm, compared to 98.0 ± 12.55 mm for *P.p.* and 121.55 ± 8.04 mm for *S.y.* The longest root length (Figure 1b) was decreased only by *P.p.*, with the control group showing a mean root length of 215.1 ± 13.35 mm, compared to 176.6 ± 19.41 mm. In contrast, the growth of seedlings from the 86 half-sib family was not affected by microbial treatments compared to the control group (C).

A two-way ANOVA was performed to assess the effects of *Family* and *Treatment* on different parameters of growth and biochemical parameters of silver birch (Table S1). For the shoot, the two-way ANOVA revealed significant main effects of Family (F(1, 152) = 19.68, *p* < 0.001) and Treatment (F(3, 152) = 3.83, *p* = 0.011). A significant interaction between *Family* and *Treatment* was also observed (F(3, 152) = 3.95, *p* = 0.010).

The analysis for root length revealed a significant main effect of *Family* (F = 15.24, df = 1, 152, *p* < 0.001), indicating that *Family* had a strong influence on root growth (Table S1).

### 2.2. Secondary Metabolism (Phenols and Flavonoids)

**Figure 2:**
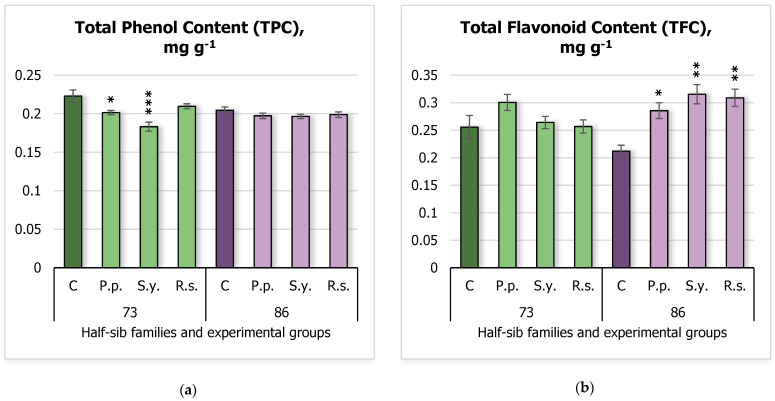
Comparison of total phenolic content (TPC) (mg g^−1^ fresh weight (FW)) (**a**) and total flavonoid content (TFC) (mg g^−1^ FW) (**b**) in silver birch half-sib families (73 and 86) under different bacterial treatments: control (C), *Pseudomonas putida* (*P.p.*), *Sphingobium yanoikuyae* (*S.y.*), and *Rhodotorula sphaerocarpa* (*R.s.*). Error bars represent the standard error (SE). Statistically significant differences compared to the control group were assessed using the Kruskal–Wallis H test: * *p* < 0.05; ** *p* < 0.01; *** *p* < 0.001.

In terms of secondary metabolism, the total phenol content (TPC) (Figure 2a) and total flavonoid content (TFC) (Figure 2b) were analyzed. The results showed that TPC decreased in seedlings of the 73 half-sib family inoculated with *P.p.* and *S.y.* bacteria, with the control group showing a mean TPC of 0.22 ± 0.01 mg g^−1^, compared to 0.20 ± 0.03 mg g^−1^ for *P.p.*, 0.18 ± 0.01 mg g^−1^ for *S.y.* In contrast, TPC in the 86 half-sib family remained unaffected. Regarding TFC, seedlings from the 86 half-sib family exhibited an increase in flavonoid content with all microbial treatments, with the control group showing a mean TFC of 0.21 ± 0.01 mg g^−1^, compared to 0.29 ± 0.01 mg g^−1^ for *P.p.*, 0.32 ± 0.02 mg g^−1^ for *S.y.*, and 0.31 ± 0.02 mg g^−1^ for *Rhodotorula sphaerocarpa* (*R.s.*), whereas the TFC of the 73 half-sib family remained unchanged compared to the control group.

The statistical analysis for TPC revealed that the treatment effect was highly significant (F = 8.16, df = 3, 64, *p* < 0.001), indicating that the different treatments had a significant impact on TPC (Table S1). Additionally, a significant interaction between *Family* and *Treatment* was observed (F = 3.76, df = 3, 64, *p* = 0.015), suggesting that the effect of *Treatment* on TPC depended on the *Family*.

The analysis for TFC showed that a significant effect of *Treatment* was observed (F = 6.50, df = 3, 57, *p* = 0.0007), suggesting that the treatments had a notable impact on TFC (Table S1). Additionally, there was a significant interaction between *Family* and *Treatment* (F = 5.17, df = 3, 57, *p* = 0.0032), indicating that the effect of *Treatment* on TFC varied depending on the *Family*.

### 2.3. Photosynthetic Pigments

**Figure 3:**
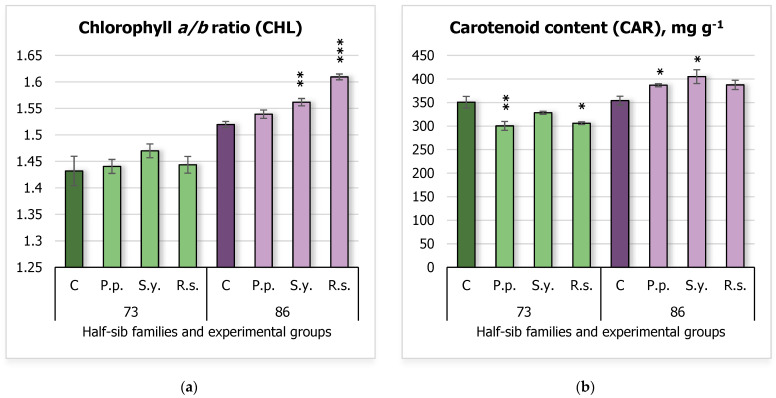
Comparison of chlorophyll *a*/*b* ratio (CHL) (**a**) and carotenoid (CAR) content (mg g^−1^ FW) (**b**) in silver birch half-sib families (73 and 86) under different bacterial treatments: control (C), *Pseudomonas putida* (*P.p.*), *Sphingobium yanoikuyae* (*S.y.*), and *Rhodotorula sphaerocarpa* (*R.s.*). Error bars represent the standard error (SE). Statistically significant differences compared to the control group were assessed using the Kruskal–Wallis H test: * *p* < 0.05; ** *p* < 0.01; *** *p* < 0.001.

Photosynthetic activity was also assessed (Figure 3) by analyzing the chlorophyll *a*/*b* (CHL) ratio (Figure 3a) and carotenoid content (CAR) (Figure 3b). The results showed that *S.y.* and *R.s.* increased the CHL ratio in seedlings of the 86 half-sib family compared to the control group. Specifically, the control group had a mean CHL of 1.52 ± 0.01, which increased to 1.56 ± 0.01 with *S.y.*, and that of 1.61 ± 0.01 with *R.s.* CAR in this family’s seedlings was also increased following inoculation with *P.p.* and *S.y.* with the control group, showing a mean CAR of 354.09 ± 9.38 mg g^−1^, compared to 386.86 ± 3.23 for *P.p.*, and 405.12 ± 14.69 mg g^−1^ for *S.y.* In contrast, seedlings from the 73 half-sib family exhibited decreased carotenoid content when inoculated with *P.p.* and *R.s.*, compared to the control group, which had a mean CAR of 350.75 ± 12.26 mg g^−1^. The CAR content decreased to 300.47 ± 9.36 with *P.p.* and 306.15 ± 2.86 with *R.s.*

Post hoc pairwise comparisons using Tukey’s adjustment for CHL revealed significant effects of *Family*, *Treatment*, and their interaction. Significant differences between family 73 and family 86 were observed across several treatments, with family 73 C differing significantly from family 86 C, *P.p.*, *R.s.*, and *S.y.* (all *p* < 0.0001). No significant differences were found between family 73 C and family 73 *P.p.*, *R.s.*, or *S.y*. For *Treatment*, family 86 C significantly differed from family 73 *P.p.*, family 73 *R.s.*, and family 86 *R.s.* (*p* = 0.0036, *p* = 0.0059, and *p* = 0.0005 respectively). Meanwhile family 73 *P.p.* showed significant differences compared with family 86 *P.p.* and 86 *R.s.* (all *p* < 0.0001). Family 86 *P.p.* was also significantly different from family 73 *R.s.*, family 86 *R.s.*, and family 73 *S.y.* (all *p* < 0.0001). The *Family* × *Treatment* interaction revealed that family 73 *R.s.* significantly differed from family 86 *R.s.* and *S.y.*, while family 86 *R.s.* differed from family 73 *S.y.* (all *p* < 0.0001). A full summary of the post hoc pairwise comparisons can be found in Table S2.

The two-way ANOVA results for CAR (Table S1) showed a significant main effect of *Family* (F = 92.05, df = 1, 64, *p* = 5.26 × 10^–14^), indicating that *Family* significantly influenced CAR levels. There was also a significant interaction between *Family* and *Treatment* (F = 9.24, df = 3, 64, *p* = 3.65 × 10^–5^), indicating that the effect of *Treatment* on CAR was dependent on the *Family*.

### 2.4. Stress Markers and Soluble Sugars

**Figure 4:**
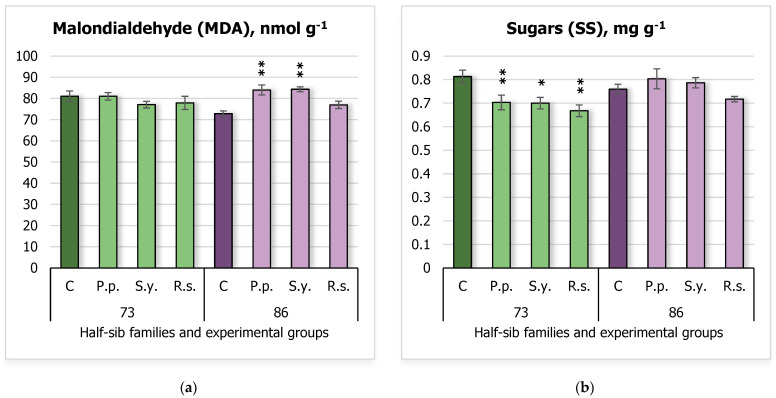
Comparison of malondialdehyde (MDA) (nmol g^−1^ FW) (**a**) and soluble sugars (SS) (mg g^−1^ FW) (**b**) in silver birch half-sib families (73 and 86) under different bacterial treatments: control (C), *Pseudomonas putida* (*P.p.*), *Sphingobium yanoikuyae* (*S.y.*), and *Rhodotorula sphaerocarpa* (*R.s.*). Error bars represent the standard error (SE). Statistically significant differences compared to the control group were assessed using the Kruskal–Wallis H test: * *p* < 0.05; ** *p* < 0.01.

Stress markers and soluble sugars were also analyzed (Figure 4), with malondialdehyde (MDA) levels presented in Figure 4a and soluble sugars in Figure 4b. The results showed that MDA levels increased in seedlings of the 86 half-sib family when inoculated with *P.p.* and *S.y*. Specifically, the control group (C) had a mean MDA level of 72.85 ± 1.28 nmol g^−1^, which increased to 83.98 ± 2.36 nmol g^−1^ with *P.p.* and 84.35 ± 1.17 nmol g^−1^ with *S.y.* In contrast, sugar (SS) levels were affected in the 73 half-sib family, with all microbial inoculations leading to decreased sugar levels compared to the control group. The control seedlings exhibited a mean SS level of 0.81 ± 0.03 mg g^−1^, which was reduced to 0.70 ± 0.03 mg g^−1^ with *P.p.*, 0.70 ± 0.02 mg g^−1^ with *S.y.*, and 0.67 ± 0.03 mg g^−1^ with *R.s.*

The two-way ANOVA results of MDA (Table S1) revealed that the *Treatment* effect was significant (F = 3.43, df = 3, 64, *p* = 0.02208), suggesting that different treatments influenced MDA levels. Additionally, the interaction between *Family* and *Treatment* was significant (F = 5.20, df = 3, 64, *p* = 0.00282), indicating that the effect of *Treatment* on MDA varied depending on the *Family*.

The results of SS indicated a significant effect of *Family* (F = 4.93, df = 1, 58, *p* = 0.0302), suggesting that *Family* influences the SS levels (Table S1). The *Treatment* effect was also significant (F = 5.06, df = 3, 58, *p* = 0.0035), indicating that different treatments led to varying levels of soluble sugars. Additionally, there was a significant interaction between *Family* and *Treatment* (F = 3.83, df = 3, 58, *p* = 0.0143), showing that the effect of *Treatment* on soluble sugars depended on the *Family*.

### 2.5. Antioxidant Enzyme Activity

**Figure 5:**
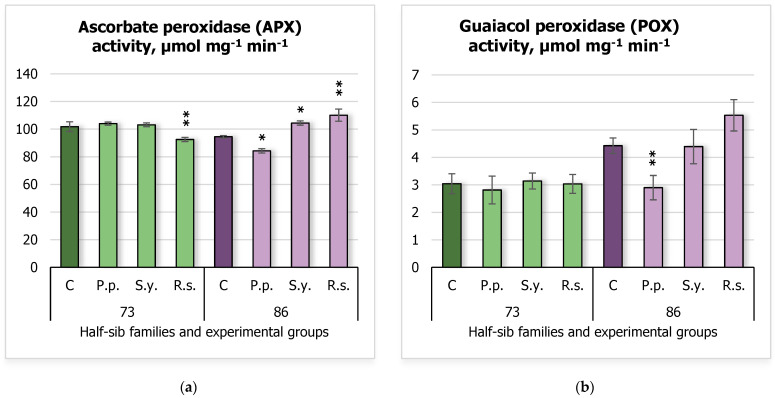
Comparison of ascorbate peroxidase (APX) activity (µmol mg^−1^ min^−1^) (**a**) and guaiacol peroxidase (POX) activity (µmol mg^−1^ min^−1^) (**b**) in silver birch half-sib families (73 and 86) under different bacterial treatments: control (C), *Pseudomonas putida* (*P.p.*), *Sphingobium yanoikuyae* (*S.y.*), and *Rhodotorula sphaerocarpa* (*R.s.*). Error bars represent the standard error (SE). Statistically significant differences compared to the control group were assessed using the Kruskal–Wallis H test: * *p* < 0.05; ** *p* < 0.01.

During the study, antioxidant enzyme activity was also measured. Regarding ascorbate peroxidase (APX) activity (Figure 5a), the 73 half-sib family seedlings showed decreased activity when inoculated with *R.s.*, with a reduction from 101.79 ± 3.51 μmol mg^−1^ min^−1^ in the control group (C) to 92.49 ± 1.54 μmol mg^−1^ min^−1^. For 86 half-sib family seedlings, APX activity decreased when inoculated with *P.p.* (94.50 ± 0.86 μmol mg^−1^ min^−1^ in the control vs. 84.26 ± 1.57 μmol mg^−1^ min^−1^ with *P.p.*) but increased when inoculated with *S.y.* (104.41 ± 1.53 μmol mg^−1^ min^−1^) and *R.s.* (110.05 ± 4.41 μmol mg^−1^ min^−1^) microorganisms. In terms of guaiacol peroxidase (POX) activity (Figure 5b), only the 86 half-sib family seedlings exhibited decreased POX activity when inoculated with *P.p.*, with values dropping from 4.43 ± 0.28 μmol mg^−1^ min^−1^ in the control to 2.90 ± 0.44 μmol mg^−1^ min^−1^.

The statistical analysis results of APX (Table S1) showed a significant effect of *Treatment* (F = 5.76, df = 3, 64, *p* = 0.00149), suggesting that different treatments led to significant changes in APX activity. Additionally, a significant interaction between *Family* and *Treatment* was found (F = 20.33, df = 3, 64, *p* < 2.28 × 10^–9^), indicating that the effect of *Treatment* on APX activity was dependent on the *Family*.

The analysis for POX revealed a highly significant effect of *Family* (F = 128.27, df = 1, 61, *p* < 2 × 10^−16^), indicating that *Family* significantly influenced POX activity (Table S1). Significant effects were also observed for *Treatment* (F = 28.06, df = 3, 61, *p* = 1.59 × 10^−11^), suggesting that the different treatments led to substantial changes in POX activity. Furthermore, the interaction between *Family* and *Treatment* was significant (F = 20.47, df = 3, 61, *p* = 2.69 × 10^−9^), demonstrating that the effect of *Treatment* on POX activity varied across *Family* groups.

**Figure 6:**
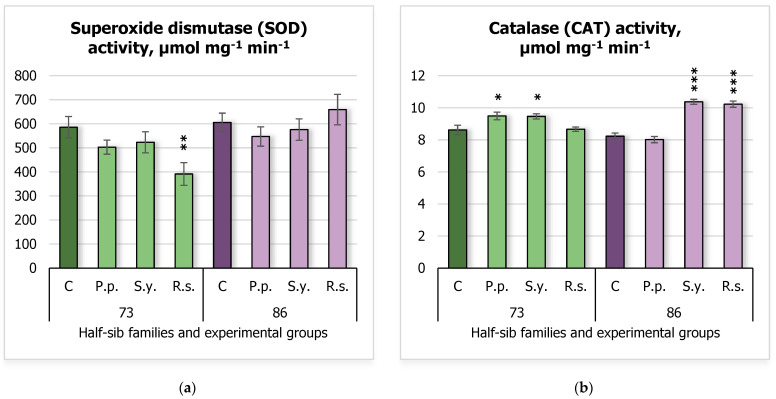
Comparison of superoxide dismutase (SOD) activity (µmol mg^−1^ min^−1^) (**a**) and catalase (CAT) activity (µmol mg^−1^ min^−1^) (**b**) in silver birch half-sib families (73 and 86) under different bacterial treatments: control (C), *Pseudomonas putida* (*P.p.*), *Sphingobium yanoikuyae* (*S.y.*), and *Rhodotorula sphaerocarpa* (*R.s.*). Error bars represent the standard error (SE). Statistically significant differences compared to the control group were assessed using the Kruskal–Wallis H test: * *p* < 0.05; ** *p* < 0.01; *** *p* < 0.001.

In terms of superoxide dismutase (SOD) activity (Figure 6a), it decreased significantly when 73 half-sib family seedlings were inoculated with yeast *R.s.*, dropping from 585.54 ± 44.48 μmol mg^−1^ min^−1^ in the control group to 391.23 ± 47.26 μmol mg^−1^ min^−1^. More significant results were observed in catalase (CAT) activity (Figure 6b). The 73 half-sib family seedlings exhibited increased CAT activity in the *P.p.* (9.49 ± 0.24 μmol mg^−1^ min^−1^) and *S.y.* (9.46 ± 0.17 μmol mg^−1^ min^−1^) experimental groups, compared to 8.62 ± 0.29 μmol mg^−1^ min^−1^ in the control. Similarly, the 86 half-sib family seedlings showed increased CAT activity when inoculated with *S.y.* (10.37 ± 0.16 μmol mg^−1^ min^−1^) and *R.s.* (10.22 ± 0.20 μmol mg^−1^ min^−1^) microorganisms, compared to 8.23 ± 0.20 μmol mg^−1^ min^−1^ in the control group.

The two-way ANOVA results of SOD (Table S1) indicated a significant effect of *Family* on SOD activity (F = 9.491, df = 1, 57, *p* = 0.00318), suggesting that different *Family* groups exhibited varying levels of SOD activity. The interaction between *Family* and *Treatment* was significant (F = 3.288, df = 3, 57, *p* = 0.02705), implying that the effect of *Treatment* on SOD activity depended on the *Family* group.

The results for CAT (Table S1) revealed that the *Treatment* factor had a significant effect on CAT activity (F = 20.003, df = 3, 62, *p* = 3.48 × 10^–9^), indicating that the different treatments led to significant changes in CAT activity. Additionally, the interaction between *Family* and *Treatment* was also significant (F = 20.253, df = 3, 62, *p* = 2.89 × 10^−9^), suggesting that the effect of *Treatment* on CAT activity was influenced by *Family*.

**Figure 7:**
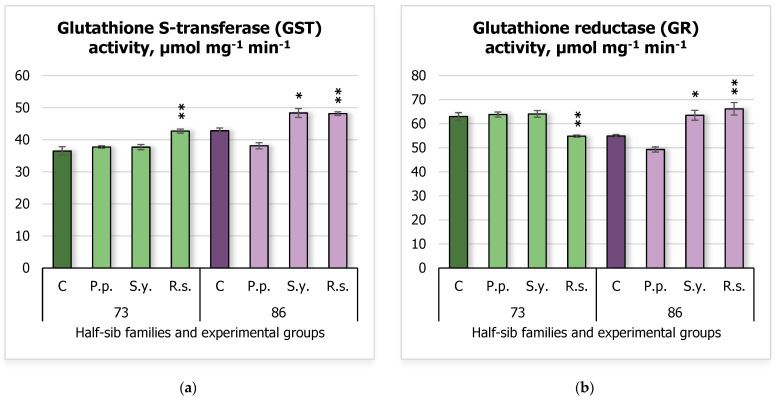
Comparison of glutathione S-transferase (GST) activity (µmol mg^−1^ min^−1^) (**a**) and glutathione reductase (GR) activity (µmol mg^−1^ min^−1^) (**b**) in silver birch half-sib families (73 and 86) under different bacterial treatments: control (C), *Pseudomonas putida* (*P.p.*), *Sphingobium yanoikuyae* (*S.y.*), and *Rhodotorula sphaerocarpa* (*R.s.*). Error bars represent the standard error (SE). Statistically significant differences compared to the control group were assessed using the Kruskal–Wallis H test: * *p* < 0.05; ** *p* < 0.01.

Results of glutathione S-transferase (GST) activity (Figure 7a) showed that *R.s.* inoculation of the 73 half-sib family resulted in increased GST activity (42.67 ± 0.63 μmol mg^−1^ min^−1^) compared to the control (36.47 ± 1.34 μmol mg^−1^ min^−1^). Additionally, the 86 half-sib family seedlings exhibited increased GST activity when inoculated with *S.y*. (48.32 ± 1.37 μmol mg^−1^ min^−1^) and *R.s.* (48.11 ± 0.56 μmol mg^−1^ min^−1^), compared to the control group (42.77 ± 0.89 μmol mg^−1^ min^−1^). Results for glutathione reductase (GR) activity indicated that the 73 half-sib family seedlings showed decreased GR activity in the *R.s.* experimental group (54.79 ± 0.45 μmol mg^−1^ min^−1^) compared to the control (62.93 ± 1.65 μmol mg^−1^ min^−1^). In contrast, the 86 half-sib family seedlings exhibited increased GR activity when inoculated with *S.y.* (63.46 ± 2.05 μmol mg^−1^ min^−1^) and *R.s.* (66.15 ± 2.60 μmol mg^−1^ min^−1^) compared to the control (54.85 ± 0.59 μmol mg^−1^ min^−1^).

The two-way ANOVA results for GST (Table S1) showed that *Family* had a significant effect on GST activity (F = 75.427, df = 1, 62, *p* = 2.59 × 10^–12^), indicating that the *Family* factor plays an important role in GST activity levels. *Treatment* also had a significant effect (F = 24.429, df = 3, 62, *p* = 1.48 × 10^–10^), suggesting that different treatments significantly altered GST activity. Furthermore, the interaction between *Family* and *Treatment* was significant (F = 9.991, df = 3, 62, *p* = 1.84 × 10^–5^), indicating that the effect of *Treatment* on GST activity was influenced by *Family*.

The results of GR (Table S1) indicated that both *Family* (F = 6.06, df = 1, 61, *p* = 0.0167) and *Treatment* (F = 7.81, df = 3, 61, *p* = 0.000171) significantly influenced GR activity. Furthermore, the interaction between *Family* and *Treatment* was highly significant (F = 26.42, df = 3, 61, *p* = 4.49 × 10^–11^), suggesting that the effect of *Treatment* on GR activity was dependent on the *Family* factor.

### 2.6. Principal Component Analysis (PCA) of Microbial Treatments Across Families

Principal component analysis (PCA) was used to investigate the impact of microbial treatments on chlorophyll *a*/*b* ratio, MDA, carotenoid, total phenolic, total flavonoid content, soluble sugars and antioxidant enzyme activity compared to the untreated control.

**Figure 8:**
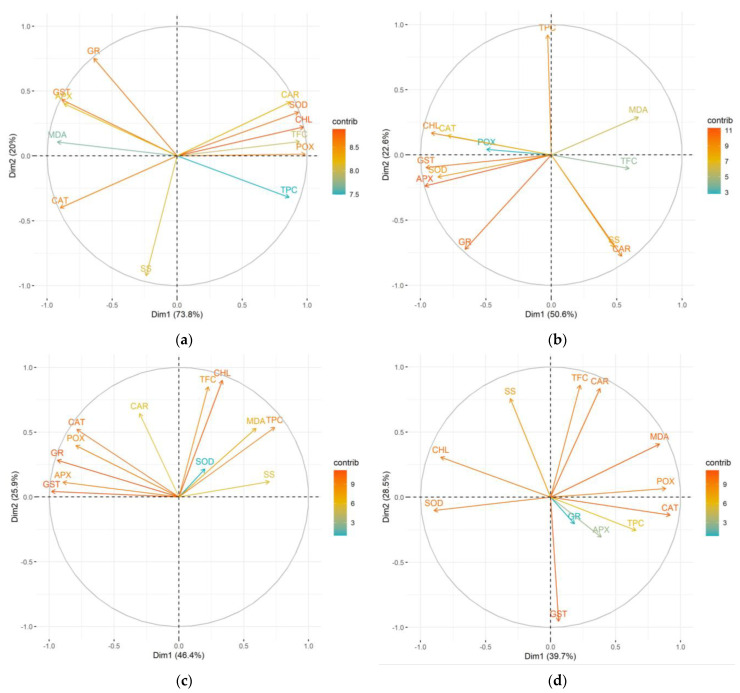
The PCA results for the phytochemical profile of silver birch half-sib family 73 across various microbial treatments: control (**a**), *Pseudomonas putida* (*P.p.*) (**b**), and *Sphingobium yanoikuyae* (*S.y.*) (**c**), and *Rhodotorula sphaerocarpa* (*R.s.*) (**d**).

In family 73, the two principal components (Dim1 and Dim2) explain 93.8% to 68.2% of all variance. In the control group (Figure 8a), Dim1 strongly positively correlates with photosynthesis pigments, phenolics, SOD and POX, while the other enzymes and MDA negatively correlate with Dim1. Dim2 is mostly determined by SS, which correlates negatively with it, as well as the GR enzyme, with a positive correlation. This dynamic changed after all three bacterial treatments. In the case of P.p. (Figure 8b), Dim1 only moderately positively correlates with CAR, TFC, SS and MDA. All the enzymes correlate strongly negatively with Dim1, as does the chlorophyll ratio. Dim2 correlates strongly positively with TPC, and negatively with GR, CAR and SS. It can be noted that *S.y.* (Figure 8c) changed the relative behavior of POX, MDA, CAR, and SS, while *R.s.* (Figure 8d) altered the dynamics altogether compared to the control group. These findings clearly showcase a strain-specific pattern in the biochemical response of the microbial treatment in this genotype/half-sib family.

**Figure 9:**
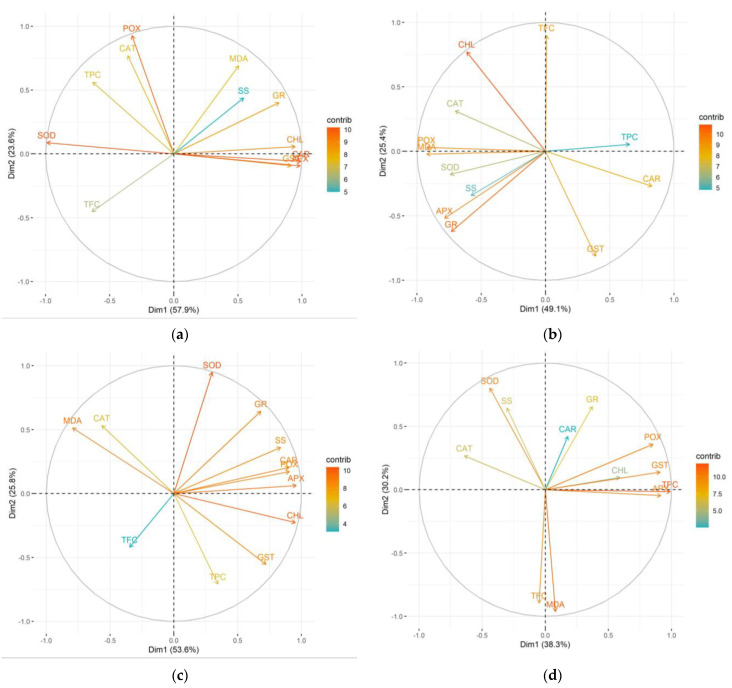
The PCA results for the phytochemical profile of the silver birch half-sib family 86 across various microbial treatments: control (**a**), *Pseudomonas putida* (*P.p.*) (**b**), *Sphingobium yanoikuyae* (*S.y.*) (**c**), and *Rhodotorula sphaerocarpa* (*R.s.*) (**d**).

In family 86, 81.5% to 68.5% of the total variance is covered by the two principal components. In Figure 9a, it can be seen that Dim1 strongly positively correlates with CHL, GST, CAR and APX. GR, SS, and MDA correlate with Dim1 too. The positions of CHL and CAR are similar to those in the other half-sib family, as are those of POX and CAT. SOD, TPC and TFC correlate moderately negatively with Dim1, while POX and CAT correlate strongly positively with Dim2. Dim2 also strongly positively correlates with MDA and moderately so with SS, while it has a positive correlation with TPC and a negative correlation with TFC. Again, after microbial treatments, this pattern changes, indicating a substantial impact on the plant’s biochemical responses, that is, as mentioned, strain-specific and birch genotype-specific. *R.s.* treatment mostly affected the relative correlations of the POX enzyme, TPC, MDA and SS, while *S.y.* affected POX, SOD, MDA and TPC. *P.p.*, on the other hand, drastically impacted TPC, POX, MDA, SS, GR, CHL, GST, APX, CAR—9 out of the 12 tested variables.

## 3. Discussion

This study investigated the impact of PAH-degrading microorganisms on the growth and biochemical responses of silver birch seedlings from two half-sib families, i.e., two genotypes, grown under hydroponic conditions. The findings highlight the family-specific responses to microbial inoculation, offering significant insights into the complex interactions between plant genotypes and microbial treatments.

The results provide compelling evidence that the response of silver birch seedlings to microbial treatments, collectively, is genotype-dependent. Family 73 experienced reduced growth and a decline in key biochemical markers, such as phenol content, carotenoids, and soluble sugars, upon microbial inoculation. Conversely, family 86 showed improved biochemical profiles, including increased flavonoid production, a higher chlorophyll *a*/*b* ratio, and enhanced antioxidant enzyme activity. These findings underscore the critical role of genetic factors in determining plant–microbe interactions, suggesting that selective breeding or the choice of specific genotypes could optimize potential application strategies. A compelling study on several tomato varieties inoculated with two *Trichoderma* species concluded with the authors remarking that “…the ability of the plant to benefit from this symbiotic-like interaction can be genetically improved…”, i.e., currently, some genotypes within the species benefit from inoculation, while others do not [29].

A broader analysis of the current study reveals distinct patterns of stress and resilience modulated by the three microbial treatments. While all tested microorganisms had variable effects, *Rhodotorula sphaerocarpa* emerged as a key differentiator. In family 86, *Rhodotorula* treatment did not increase malondialdehyde (MDA) levels, a marker of oxidative stress, while it also prevented significant growth reductions in both families. These results indicate that microbial species and their functional roles must be carefully considered to maximize benefits in stress alleviation [30,31]. While the *Rhodotorula sphaerocarpa* species has not been thoroughly investigated previously, other species from the genus have been shown to induce systemic resistance in drought-affected tomatoes, increasing levels of antioxidant enzymes and phenolics, and increasing plant weight and biomass [32]. Furthermore, previous studies have indicated that *R. sphaerocarpa* has a positive impact on black alders (*Alnus glutinosa* L.) in terms of growth enhancement [33].

The negative effects observed in family 73 raise important questions about the mechanisms driving these interactions. The reduced levels of biochemical markers like phenolics and carotenoids, compounds known for their stress-mitigating properties, suggest that microbial inoculation could disrupt certain metabolic pathways in susceptible genotypes, as the opposite was shown to be possible [34].

This contrasts the case for family 86, where enhanced flavonoid production and antioxidant enzyme activities suggest that the microorganisms successfully induced systemic resistance [35,36,37], supporting the hypothesis that PAH-degrading microorganisms could likely alleviate stress.

These findings align partially with the findings in the existing literature on plant–microbe interactions under stress conditions. PAH-degrading microorganisms, including *Pseudomonas* spp. and *Sphingobium* spp., are well documented for their ability to not only degrade pollutants directly [18,38,39,40] but also to enhance plant growth by modulating biochemical pathways. For instance, *Pseudomonas putida* was shown to improve *Populus*, *Arabidopsis* and cucumber growth and health [41,42,43]. Similarly, *Sphingobium yanoikuyae* has also been reported to improve growth in rice [44] and increase cadmium tolerance in *Salix* trees [20]. These are qualities that would help in aiding plant resilience under stressful conditions.

However, the family-specific responses observed in this study introduce nuances to these general findings. While most previous studies focus on the benefits of microbial inoculation [23,24,25,26,27], this research highlights potential trade-offs, such as growth inhibition and reduced metabolic activity in susceptible genotypes like family 73. These results echo findings from studies on genotype-dependent microbial interactions, where certain plant genotypes fail to establish beneficial symbioses, potentially due to incompatible signaling pathways or resource competition [30,45].

This study fills a critical knowledge gap by demonstrating that the effects of PAH-degrading microorganisms on tree species are not universally positive but are contingent on genotype. The observed biochemical changes suggest that microbial inoculation can influence metabolic pathways involved in stress response and growth regulation. For instance, the increased flavonoid production and antioxidant enzyme activities in family 86 may indicate the activation of systemic acquired resistance (SAR), a well-known mechanism in plant–microbe interactions [16,17,18].

The contrasting responses between the two families also raise questions about the underlying genetic and molecular mechanisms. It is possible that family 73 lacks key genes or regulatory elements necessary for establishing beneficial interactions with these microorganisms [30,45]. Alternatively, the observed growth inhibition and reduced biochemical marker levels in family 73 may result from microbial-induced resource competition or maladaptive hormonal responses, such as altered auxin [46] or ethylene signaling [47]. While this study provides valuable insights, several limitations should be acknowledged. First, the hydroponic setup, while useful for controlled experiments, does not fully replicate the complexity of soil-based systems [48]. Soil factors, such as nutrient availability, pH, and microbial community composition, could significantly influence plant–microbe interactions in real-world conditions. Second, the focus on three specific microorganisms, though informative, limits the generalizability of the findings. Future studies should explore a broader range of microbial species and consortia to identify the most effective species/combinations for inducing resistance [49]. Additionally, the genetic basis of the observed family-specific responses remains unexplored. Integrating omic analyses could provide deeper insights into the molecular mechanisms underlying these interactions [50]. Long-term effects of microbial inoculation on tree growth, health, and pollutant degradation under field conditions should also be considered [51,52]. This would provide a more comprehensive understanding of the practical implications of these interactions.

## 4. Materials and Methods

During the study, two half-sib families (individuals sharing one common parent) of silver birch (*Betula pendula* Roth.) seeds, numbered 73 and 86, were inoculated with three microorganisms known for their ability to degrade PAHs: *Pseudomonas putida* Trevisan (*P.p.*), *Sphingobium yanoikuaye* Yabuuchi et al. (*S.y.*), and *Rhodotorula sphaerocarpa* (S.Y. Newell and Fell) Q.M. Wang, F.Y. Bai, M. Groenewald and Boekhout (*R.s.*). The seedlings were grown in hydroponic conditions, and after 4 weeks, their morphological and biochemical parameters were evaluated to assess the impact of PAH-degrading microorganisms on silver birch growth. The seeds of silver birch were collected in 2024 from trees in the second-generation seed plantation at Dubrava Regional Park, Vaisvydava Forest, Lithuania (coordinates—54°51′20.6″ N 24°03′04.8″ E; 70.57 M.A.S.L.).

### 4.1. Inoculation of Seeds with PAH-Degrading Microorganisms, Experimental Plant Preparation and Hydroponic Setup

Three microorganisms capable of degrading PAHs were chosen for the study due to their safety for humans and plants and their capacity to colonize plant tissues. These included *Pseudomonas putida* Trevisan (*P.p.*)—DSM No. 28064—and *Sphingobium yanoikuyae* Yabuuchi et al. (*S.y.*)—DSM No. 6900—both sourced from The Leibniz Institute DSMZ, as well as *Rhodotorula sphaerocarpa* (S.Y. Newell and Fell) Q.M. Wang, F.Y. Bai, M. Groenewald, and Boekhout (*R.s.*)—MUCL No. 030605—obtained from the Belgian Coordinated Collections of Microorganisms (BCCM).

The inoculation of silver birch (*Betula pendula* Roth.) seeds and preparation of seedlings followed the protocol described by Striganavičiūtė et al. [33], with modifications. Briefly, microorganisms were cultured in liquid LB medium, harvested, and washed in 0.9% NaCl solution. For inoculation, 2000 silver birch seeds were soaked in 40 mL of the microorganism suspension, with the OD of the suspension set to 1 at 600 nm, for 30 min, with occasional mixing. Control seeds were treated with 40 mL of 0.9% NaCl solution under the same conditions. After soaking, seeds were air-dried and planted in glass Petri dishes lined with filter paper moistened with tap water. After one week of germination, the seedlings were transferred to rockwool cubes previously prepared by soaking them in distilled water adjusted to pH 5.6, using 0.1–1 N HCl and NaOH solutions (both from Duchefa Biochemie, Haarlem, The Netherlands). The cubes were then irrigated with modified full-strength (100%) Hoagland’s nutrient solution, which contained 6 mM KNO_3_, 2.32 mM Ca(NO_3_)_2_ × 4 H_2_O, 1.86 mM MgSO_4_ × 7 H_2_O, 1 mM NH_4_H_2_PO_4_, 46 μM H_3_BO_3_, 9 μM MnCl_2_ × 4 H_2_O, 8.99 μM C_12_H_12_Fe_2_O_18_, 0.76 μM ZnSO_4_ × 7 H_2_O, 0.5 μM CuSO_4_ × 5 H_2_O, and 0.58 μM Na_2_MoO_4_ × 2 H_2_O.

The trays containing the rockwool cubes and seedlings were incubated under controlled conditions. Initially, they were kept in the dark for one week at a day/night temperature of 25 °C/20 °C, after which they were transferred to a light environment with an irradiance of 94.5 μmol m^−2^ s^−1^. Covers were removed, and the seedlings were watered as needed with half-strength (50%) Hoagland’s nutrient solution. Seedlings were maintained under these conditions for four weeks, until reaching the BBCH14 growth stage [53].

For the hydroponic system, 10 L black plastic containers (30 cm × 25 cm × 12 cm) were employed, with 2 cm thick polystyrene foam boards used as covers. A plastic netting panel, designed to fit the containers, was inserted to hold the rockwool cubes in place. Continuous aeration was provided by air pumps (Union Star Air AC-500, 230 V, 50 Hz, 2 W, China) connected to plastic tubing, non-return valves (12 × 7 × 3 cm), and 2 cm air pebbles. The seedlings were grown in a growth chamber for a period of four weeks.

After four weeks, morphometric parameters, including shoot and root length, were measured. The mean shoot length was calculated at the beginning and end of the experiment to assess growth, while root length was measured only at the end. Leaves were harvested for subsequent biochemical analysis.

### 4.2. Biochemical Analysis

#### 4.2.1. Extract Preparation for Photosynthetic Pigments, Secondary Metabolites, MDA, and Sugar Analysis

For each group, 3 × 0.1 g of collected leaves were ground using a “Precellys 24” tissue homogenizer (Bertin Technologies, Montigny-le-Bretonneux, France) at 1956× *g* for 30 s with two metal beads. Afterward, 1.5 mL of 80% ethanol (*v*/*v* in water, MV GROUP Production, Vilnius, Lithuania) was added, and the mixture was homogenized again at 1956× *g* for 30 s. The mixture was then centrifuged at 21,910× *g* for 30 min at 4 °C using a Hettich Universal 32R centrifuge (Andreas Hettich GmbH and Co. KG, Tuttlingen, Germany). The supernatant was collected and used for the analysis of chlorophyll, carotenoids, secondary metabolites, malondialdehyde, and soluble sugars.

#### 4.2.2. Total Phenolic Content (TPC) Measurement

TPC was measured using a modified Lowry method with Folin–Ciocalteu reagent. The extract was combined with Folin–Ciocalteu reagent (1:9 *w/v* in water) and incubated for 5 min. Sodium carbonate (Na_2_CO_3_) was then added, and the mixture was kept in the dark for 1 h. Absorbance was recorded at 725 nm using a microplate reader. For detailed procedures, refer to the publication by Čėsnienė et al. [54].

#### 4.2.3. Total Flavonoid Content (TFC) Measurement

TFC was determined based on the method outlined by Chang et al. [55]. Absorbance measurements were taken at 415 nm. The extract was combined with a reaction buffer containing absolute ethyl alcohol (MERCK, Darmstadt, Germany), aluminum chloride (Alfa Aesar, Karlsruhe, Germany), potassium acetate (Sigma Aldrich, St. Louis, MO, USA), and distilled water. Detailed steps and calculations are provided in Čėsnienė et al. [54] publication.

#### 4.2.4. Chlorophyll a and b, and Carotenoid Measurement

Absorbance of the extract was measured at 470, 648, and 664 nm using a SpectroStar Nano microplate reader (BMG Labtech, Offenburg, Germany) and 96-well microplates. The formulas and models for analysis were as described in Čėsnienė et al. [54].

#### 4.2.5. Malondialdehyde (MDA) Measurement

MDA was measured by mixing the supernatant with a reaction mixture containing trichloroacetic acid (Molar chemicals Kft, Budapest, Hungary) and thiobarbituric acid (Alfa Aesar, Karlsruhe, Germany) according to Čėsnienė et al. [54]. The mixture was incubated at 95 °C for 30 min, then cooled on ice. Absorbance was measured at 440, 532, and 600 nm.

#### 4.2.6. Soluble Sugars (SS) Measurement

Soluble sugars were determined by mixing the sample with anthrone reagent (Carl Roth, Karlsruhe, Germany). The reagent was prepared by dissolving anthrone in concentrated H_2_SO_4_ (Chempur, Piekary Śląskie, Poland), following Čėsnienė et al. [54]. The mixture was incubated at 90 °C for 1 h, and absorbance was recorded at 620 nm.

#### 4.2.7. Potassium Phosphate Buffer Preparation

K-phosphate buffers with different pH values were prepared by mixing stock solutions of 1 M K_2_HPO_4_ (Carl Roth, Germany) and 1 M KH_2_PO_4_ (Chempur, Poland). Buffers with pH values of 6.5, 7, and 7.8 were prepared as needed.

#### 4.2.8. Enzyme Extract Preparation

For enzyme activity assays, fresh biomass (3 × 0.1 g per group) was ground with liquid nitrogen and mixed with an extraction buffer containing K-phosphate buffer (pH 7.8), Triton-X, polyvinylpolypyrrolidone (PVPP), and ascorbic acid (ASC). The mixture was centrifuged at 21,910× *g* and −4 °C for 1 h, and the supernatant was used to measure protein levels, catalase (CAT), and superoxide dismutase (SOD) activity. For ascorbate peroxidase (APX), guaiacol peroxidase (POX), glutathione reductase (GR), and glutathione S-transferase (GST), the supernatant was further purified using Sephadex G-25 columns (Column PD-10, Cytiva, Gillingham, UK) as described by Kozlowski, Buchala, and Métraux [56]. All procedures were performed on ice to maintain sample integrity.

#### 4.2.9. Total Protein (PROT) Quantification

Protein concentration was determined by mixing the crude extract with Biuret reagent, which contains CuSO_4_, Na-K tartrate, Na_2_CO_3_ in NaOH solution, and Folin–Ciocalteu reagent. After incubation at room temperature, absorbance was measured at 660 nm. Protein concentrations were expressed as micrograms of Bovine Serum Albumin (BSA) equivalent per milliliter of crude extract.

#### 4.2.10. Catalase Enzyme (CAT) Activity Measurement

Catalase activity was assessed by mixing the extract with K-phosphate buffer (pH 7) and hydrogen peroxide (H_2_O_2_) solution. Absorbance was measured at regular intervals to monitor the activity, as described by Čėsnienė et al. [54].

#### 4.2.11. Superoxide Dismutase Enzyme (SOD) Activity Measurement

SOD activity was evaluated by combining the extract with a reaction buffer consisting of K-phosphate buffer (pH 7.8), methionine, nitro blue tetrazolium (NBT), ethylenediamine tetraacetic acid (EDTA), and riboflavin. The reaction was exposed to white light, and absorbance was measured at 550 nm to determine SOD activity, according to Čėsnienė et al. [54].

#### 4.2.12. Guaiacol Peroxidase Enzyme (POX) Activity Measurement

POX activity was assessed by mixing the extract with a reaction solution containing pyrogallol, a 50 mM K-phosphate buffer (pH 6.5), and 10% hydrogen peroxide (H_2_O_2_). Absorbance at 430 nm was measured at intervals to monitor activity, as outlined by Čėsnienė et al. [54].

#### 4.2.13. Ascorbate Peroxidase Enzyme (APX) Activity Measurement

APX activity was measured by combining the extract with ascorbic acid (ASC) solution and 10% hydrogen peroxide (H_2_O_2_). Absorbance at 290 nm was recorded to track the decrease over time, following the procedure of Čėsnienė et al. [54].

#### 4.2.14. Glutathione S-Transferase Enzyme (GST) Activity Measurement

GST activity was measured by mixing the extract with a reaction buffer containing CDNB and GSH. Absorbance at 340 nm was measured, and enzyme activity was calculated based on the change in absorbance over time, as described by Striganavičiūtė et al. [57].

#### 4.2.15. Glutathione Reductase Enzyme (GR) Activity Measurement

To measure GR enzyme activity, the extract was combined with a reaction buffer consisting of HEPES buffer (pH 8), EDTA, and NADPH. After adding GSSG, absorbance changes at 340 nm were monitored over time, according to Čėsnienė et al. [54].

### 4.3. Principal Component Analysis (PCA)

Principal component analysis (PCA) was performed on a numeric dataset containing various traits across microbial treatments and family groups. Missing values were removed using the *na.omit()* function. Non-numeric columns, such as identifiers, were excluded, and the remaining data were standardized to have a mean of zero and a standard deviation of one using the *scale()* function. PCA was then conducted using the *prcomp()* function, with centering and scaling applied. This approach reduced the dimensionality of the data while retaining the maximum variance, and a summary of the PCA results was generated to assess the variance explained by each principal component.

To visualize the results, a scree plot was created to display the variance explained by each principal component using the *fviz_eig()* function. A PCA plot of individuals was generated to visualize the distribution of data points along the principal components, with confidence ellipses (95% level) added using the *fviz_pca_ind()* function. Additionally, a PCA plot of variables was created to highlight the contribution of each variable to the principal components, using the *fviz_pca_var()* function.

All analyses were conducted using R (version 4.3.1, R Foundation for Statistical Computing, [Vienna, Austria]), with the *factoextra* and *ggplot2* packages used for PCA and visualization.

### 4.4. Statistical Analysis

The data were organized, and graphs were created using Microsoft Office Excel. Statistical analyses were performed using SPSS version 28.0.1.1 (IBM Inc.), where the Kruskal–Wallis H test was applied for independent samples as a non-parametric substitute for one-way ANOVA. Pairwise comparisons of ranks were conducted using Dunn’s test (*p*  <  0.05). A confidence level of 95% was maintained. The results of Dunn’s test were reported with adjusted *p*-values (Bonferroni correction) to highlight the specific groups that showed significant differences.

A two-way ANOVA was performed in R to analyze the effects of Family and Treatment on different tested parameters (except chlorophyll *a*/*b* ratio). The model, a type of general linear model, included the interaction term (Family × Treatment) and was fitted using the *aov()* function. Assumptions of homogeneity of variances were assessed using Levene’s test (*leveneTest()* from the *car* package), and all variances were confirmed to be homogeneous.

The effect of Family, Treatment, and their interaction on chlorophyll content (CHL) was analyzed using Welch’s ANOVA due to violations of the homogeneity of variance assumption. Prior to analysis, the assumption of homogeneity of variances was tested using Levene’s test (*LeveneTest* function from the *car* package). In cases where Levene’s test indicated a significant heterogeneity of variances, Welch’s ANOVA was applied to account for unequal variances among groups. The main effects of Family and Treatment, as well as their interaction, were examined, and pairwise comparisons were performed using the *emmeans* package with Tukey’s adjustment for multiple comparisons.

## 5. Conclusions

In conclusion, this study highlights the intricate relationship between plant genotype and microbial inoculation, demonstrating that PAH-degrading microorganisms can significantly influence the growth and biochemical responses of *Betula pendula* seedlings in a genotype-dependent manner. Notably, inoculation with yeast *Rhodotorula sphaerocarpa* induced systemic resistance in half-sib family 86, suggesting a strong genotype–microbe interaction that modulates plant stress responses. These findings emphasize the need to consider genetic variability when applying microbial treatments to enhance plant resilience. By shedding light on both the benefits and constraints of microbial inoculation, this research advances our understanding of plant–microbe interactions and their role in stress adaptation. Beyond environmental applications, these insights contribute to the broader fields of plant physiology and microbial ecology, with potential implications for improving tree health and growth in challenging conditions. Future studies should further explore the molecular mechanisms underlying these interactions to optimize microbial applications for plant health and development.

## Data Availability

The data supporting the reported results are available upon reasonable request from the corresponding author. Due to privacy and ethical restrictions, the raw data cannot be publicly archived.

## Supplementary Materials

The following supporting information can be downloaded at https://www.mdpi.com/article/10.3390/plants14040545/s1. Table S1: Results of two-way ANOVA for growth and biochemical parameters of silver birch inoculated with different microorganisms. Significance: * *p* ≤ 0.05; ** *p* ≤ 0.01; *** *p* ≤ 0.001; Table S2: Post hoc pairwise comparisons of Family and Treatment effects on CHL with an SE value of 0.0196. Significant differences are indicated by *p*-values less than 0.05.

## Abbreviations

The following abbreviations are used in this manuscript:

APX	Ascorbate Peroxidase
C	Control
CAR	Carotenoid content
CAT	Catalase
CHL	Chlorophylls *a*/*b* ratio
GR	Glutathione Reductase
GST	Glutathione S-transferase
MDA	Malondialdehyde
*P.p.*	*Pseudomonas putida*
PAH	Polycyclic Aromatic Hydrocarbons
PCA	Principal Component Analysis
POX	Guaiacol Peroxidase
PROT	Total Proteins
*R.s.*	*Rhodotorula sphaerocarpa*
*S.y.*	*Sphingomonas yanoikuyae*
SOD	Superoxide Dismutase
SS	Soluble Sugars
TFC	Total Flavonoid Content
TPC	Total Phenolic Content
