# Supplementary material for: Microbial Allies or Adversaries? The Genotype-Dependent Impact of Inoculation on Silver Birch
Source: Plants (Basel). 2025 Feb 10;14(4):545. doi: 10.3390/plants14040545 (PMC11859868; doi:10.3390/plants14040545)
Supplement: Supplementary file 1 [file plants-14-00545-s001.zip › plants-3458179-supplementary.pdf]

plants

Supplementary materials

Microbial Allies or Adversaries? The Genotype-Dependent Impact of Inoculation on Silver Birch

Greta Striganavičiūtė *, Dorotėja Vaitiekūnaitė, Milana Šilanskienė and Vaida Sirgedaitė-Šėžienė

Laboratory of Forest Plant Biotechnology, Institute of Forestry, Lithuanian Research Centre for Agriculture and Forestry, LT-53101 Kaunas, Lithuania; doroteja.vaitiekunaite@lammc.lt (D.V.); milana.silanskiene@lammc.lt (M.Š.); vaida.seziene@lammc.lt (V.S.-Š.)

* Correspondence: greta.striganaviciute@lammc.lt

Table S1. Results of Two-Way ANOVA for Growth and Biochemical Parameters of Silver Birch Inoculated with Different Microorganisms. Significance: *$p \leq 0.05$; **$p \leq 0.01$; ***$p \leq 0.001$

Parameter	Factor	dF	Sum Sq	Mean Sq	F value	*p*-value
Shoot growth	Family	1	41570	41570	19.68	**<0.001** ***
	Microbial Treatment	3	24290	8097	3.83	**0.011** *
	Family × Treatment	3	25003	8334	3.95	**0.010** **
	Residual	152	321058	2112		
Root length	Family	1	105781	105781	15.243	**0.000142** ***
	Microbial Treatment	3	44701	105781	2.147	0.096644
	Family × Treatment	3	14755	4918	0.709	0.548210
	Residual	152	1054827	6940		
Total Phenolic Content (TPC)	Family	1	0.000445	0.0004449	2.039	0.158178
	Microbial Treatment	3	0.005338	0.0017794	8.155	**0.000112** ***
	Family × Treatment	3	0.002460	0.0008200	3.758	**0.015003** *
	Residual	64	0.013964	0.0002182		
Total Flavonoid Content (TFC)	Family	1	0.00319	0.003190	1.848	0.179328
	Microbial Treatment	3	0.03363	0.011210	6.496	**0.000739** ***
	Family × Treatment	3	0.02674	0.008915	5.166	**0.003153** **
	Residual	57	0.09836	0.001726		
Carotenoid content (CAR)	Family	1	69153	69153	92.050	**5.26×10^{-14}** ***
	Microbial Treatment	3	5607	1869	2.488	0.0684
	Family × Treatment	3	20832	6944	9.243	**3.65×10^{-5}** ***
	Residual	64	48080	751		
	Family	1	1.3	1.26	0.034	0.85484

Malondialdehyde (MDA)	Microbial Treatment	3	383.0	127.68	3.432	**0.02208** *
	Family × Treatment	3	580.2	193.39	5.199	**0.00282** **
	Residual	64	2380.7	37.20		
Sugars (SS)	Family	1	0.02589	0.025886	4.934	**0.0302** *
	Microbial Treatment	3	0.07966	0.026554	5.061	**0.0035** **
	Family × Treatment	3	0.06025	0.020083	3.828	**0.0143** *
	Residual	58	0.30428	0.005246		
Ascorbate peroxidase (APX)	Family	1	74	74.3	1.371	0.24604
	Microbial Treatment	3	938	312.5	5.764	**0.00149** **
	Family × Treatment	3	3307	1102.2	20.331	**2.28×10^{-9}** ***
	Residual	64	3470	54.2		
Guaiacol peroxidase (POX)	Family	1	28.11	28.110	128.27	**$< 2 \times 10^{-16}$** ***
	Microbial Treatment	3	18.45	6.150	28.06	**1.59×10^{-11}** ***
	Family × Treatment	3	13.46	4.487	20.47	**2.69×10^{-9}** ***
	Residual	61	13.37	0.219		
Superoxide dismutase (SOD)	Family	1	147236	147236	9.491	**0.00318** **
	Microbial Treatment	3	53696	17899	1.154	0.33540
	Family × Treatment	3	153006	51002	3.288	**0.02705** *
	Residual	57	884269	15513		
Catalase (CAT)	Family	1	0.204	0.204	0.515	0.475
	Microbial Treatment	3	23.787	7.929	20.003	**3.48×10^{-9}** ***
	Family × Treatment	3	24.085	8.028	20.253	**2.89×10^{-9}** ***
	Residual	62	24.576	0.396		
Glutathione-S-transferase (GST)	Family	1	581.2	581.2	75.427	**2.59×10^{-12}** ***
	Microbial Treatment	3	564.7	188.2	24.429	**1.48×10^{-10}** ***
	Family × Treatment	3	231.0	77.0	9.991	**1.84×10^{-5}** ***
	Residual	62	477.8	7.7		
	Family	1	126.0	126.0	6.06	**0.016668** *

	Microbial Treatment	3	487.2	162.4	7.81	**0.000171 *****
Glutathione reductase (GR)	Family × Treatment	3	1647.9	549.3	26.42	**4.49×10^{-11} *****
	Residual	61	1268.3	20.8		

Table S2. Post hoc pairwise comparisons of Family and Treatment effects on CHL with SE value of 0.0196. Significant differences are indicated by *p*-values less than 0.05.

Contrast	Estimate	t.ratio	*p*-value
Family73 C - Family86 C	-0.08761	-4.467	**0.0008**
Family73 C - Family73 *P.p.*	-0.00859	-0.438	0.9998
Family73 C - Family86 *P.p.*	-0.10720	-5.465	**<0.0001**
Family73 C - Family73 *R.s.*	-0.01164	-0.593	0.9988
Family73 C - Family86 *R.s.*	-0.17758	-9.054	**<0.0001**
Family73 C - Family73 *S.y.*	-0.03803	-1.939	0.5303
Family73 C - Family86 *S.y.*	-0.12988	-6.622	**<0.0001**
Family86 C - Family73 *P.p.*	0.07902	4.029	**0.0036**
Family86 C - Family86 *P.p.*	-0.01958	-0.998	0.9731
Family86 C - Family73 *R.s.*	0.07597	3.874	**0.0059**
Family86 C - Family86 *R.s.*	-0.08996	-4.587	**0.0005**
Family86 C - Family73 *S.y.*	0.04959	2.528	0.2026
Family86 C - Family86 *S.y.*	-0.04226	-2.155	0.3925
Family73 *P.p.*- Family86 *P.p.*	-0.09861	-5.028	**0.0001**
Family73 *P.p.*- Family73 *R.s.*	- 0.00305	-0.156	1.0000
Family73 *P.p.* - Family86 *R.s.*	- 0.16899	-8.616	**<0.0001**

Family73 *P.p.* - Family73 *S.y.*	- 0.02944	- 1.501	0.8041
Family73 *P.p.* - Family86 *S.y.*	-0.12129	-6.184	**<0.0001**
Family86 *P.p.* - Family73 *R.s.*	0.09556	4.872	**0.0002**
Family86 *P.p.* - Family86 *R.s.*	-0.07038	-3.588	**0.0141**
Family86 *P.p.* - Family73 *S.y.*	0.06917	3.527	**0.0169**
Family86 *P.p.* - Family86 *S.y.*	-0.02268	- 1.156	0.9411
Family73 *R.s.* - Family86 *R.s.*	-0.16594	-8.460	**<0.0001**
Family73 *R.s.* - Family73 *S.y.*	-0.02639	-1.345	0.8777
Family73 *R.s.* - Family86 *S.y.*	-0.11824	-6.028	**<0.0001**
Family86 *R.s.* - Family73 *S.y.*	0.13955	7.115	**<0.0001**
Family86 *R.s.* - Family86 *S.y.*	0.04770	2.432	0.2441
Family73 *S.y.* - Family86 *S.y.*	-0.09185	-4.683	**0.0004**
